# The long noncoding RNA LINC00312 induces lung adenocarcinoma migration and vasculogenic mimicry through directly binding YBX1

**DOI:** 10.1186/s12943-018-0920-z

**Published:** 2018-11-23

**Authors:** Zhenzi Peng, Jun Wang, Bin Shan, Bin Li, Wei Peng, Yeping Dong, Wenwen Shi, Wenyuan Zhao, Dan He, Minghao Duan, Yuanda Cheng, Chunfang Zhang, Chaojun Duan

**Affiliations:** 1 Institute of Medical Sciences, Key Laboratory of Cancer Proteomics of Chinese Ministry of Health, Xiangya Hospital, Central South University, Xiangya Road 87th, Changsha, 410008 Hunan People’s Republic of China; 2 Elison S Floyd College of Medicine, Washington State University, Spokane, WA 99201 USA; 3 Department of Thoracic Surgery, Xiangya Hospital, Central South University, Changsha, 410008 People’s Republic of China; 4 Hunan Cancer Hospital, The Affiliated Tumor Hospital of Xiangya Medical College, Central South University, Changsha, 410008 People’s Republic of China

**Keywords:** LINC00312, YBX1, Metastasis, Vasculogenic mimicry, Lung adenocarcinoma

## Abstract

Vasculogenic mimicry (VM) gives rise to tumor neovascularization that is critical for tumor growth and metastasis. Long non-coding RNAs (lncRNAs) have been implicated in diverse and fundamental biological processes. LINC00312 is associated with lung adenocarcinoma. In this study, we found that LINC00312 induced migration, invasion and VM of lung cancer cells by direct binding to the transcription factor Y-Box Binding Protein 1 (YBX1). Moreover, we demonstrated that YBX1 is associated with different fragments within 0–2410 nt 5’region of LINC00312. In addition, LINC00312 is associated with VM in 124 lung adenocarcinoma clinical specimens. The results suggest that LINC00312 is a promising therapeutic and diagnostic target for lung adenocarcinoma.

**Electronic supplementary material:**

The online version of this article (10.1186/s12943-018-0920-z) contains supplementary material, which is available to authorized users.

## Main text

Lung cancer is the most common cause of cancer related death in men and women. One of the most common types of lung cancer is NSCLC. Tumor neovascularization provides adequate blood and nutrition supply for tumor progression. Vasculogenic mimicry (VM) is one of the important sources of angiogenesis and nutritional supply in cancer [1]. The lncRNA metastasis-associated lung adenocarcinoma transcript 1 (MALAT-1) promotes the formation of VM in vitro, which suggests a role of lncRNA in angiogenesis [2]. We profiled lncRNA expression in lung adenocarcinoma (ADC) and identified LINC00312 as a differentially expressed lncRNA in our previous work [3, 4]. However, whether LINC00312 associated with VM in ADC remains unclear.

### Higher expression of LINC00312 was associated with metastasis of ADC patients

**Fig. 1:**
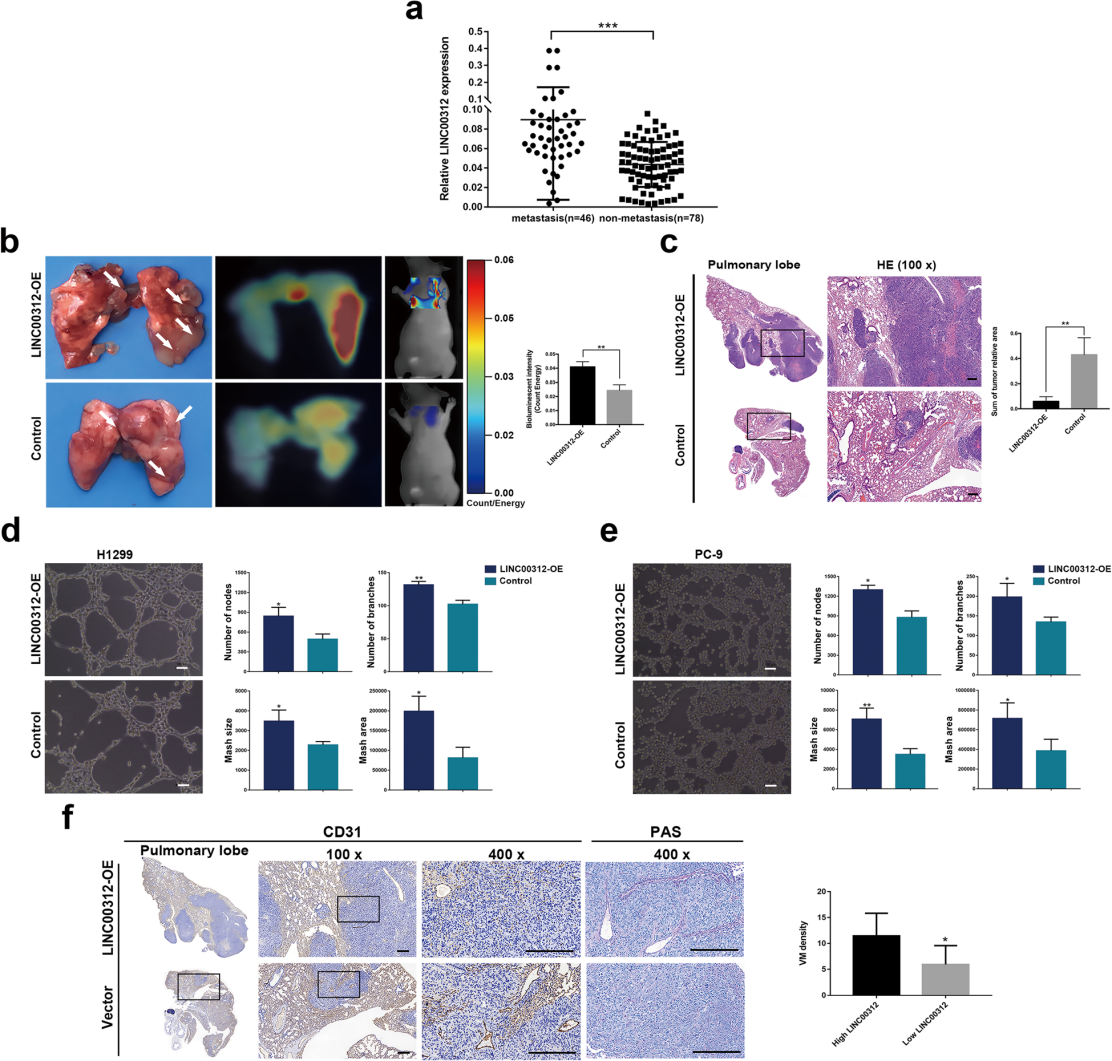
Ectopic expression of LINC00312 promoted metastasis and VM of ADC cells. **a** Boxplot of LINC00312 expression from 124 ADC patients. High LINC00312 expression was positively correlated with TNM metastasis. **b** The quantitation of lung metastasis was assessed by bioluminescence measurements. The bioluminescent change in LINC00312-OE group was significantly increased compared with the control. **c** Hematoxylin and eosin (HE) staining in the lung samples obtained from mice after injection with stable pIRES2-LINC00312 PC-9 cells. **d** and **e** Schematic presentation of the ability of ADC cells form VMs. Bar charts showing number of nodes, number of branches, Mash size and Mash area in stable. Quantification from three independent replicates is shown on the right. **f** Typical images of CD31-/PAS+ double staining in the lung samples obtained from mice after injection with stable pIRES2-LINC00312 PC-9 cells. Control represent wild cells transfected with vector plasmid. Error bars represent SD from three reactions. Significance was calculated using Student’s t test. *p < 0.05; **p < 0.001; ***p < 0.0001; ns, not significant. Scale bars: (**c**, **d**, **e**)100μm in 100 x images, (**f**) 200μm in 100x and 400x images

In this study, we measured the expression level of LINC00312 in 124 paired ADC tumor tissues and its adjacent non-tumor lung tissues using qRT-PCR. LINC00312 expression in patients with ADC metastasis was higher than patients without metastasis (*p* < 0.0001) (Fig. 1a). The clinicopathological analysis revealed that LINC00312 high expression was associated with lymph node metastasis (*p* < 0.001), distant metastasis (*p* < 0.001), tumor node metastasis (TNM) stage (*p* = 0.042) (Additional file 1: Table S1).

### Ectopic expression of LINC00312 promoted metastasis and VM of ADC cells

To further characterize the role of LINC00312, we measured metastasis of ADC cells in vivo using bioluminescence imaging. The mice inoculated with the pIRES2-LINC00312 PC-9 overexpression cells exhibited more lung metastatic tumor nodules than the mice inoculated with the control PC-9 cells (Fig. 1b). Increased metastasis to the lung was confirmed by histological analysis. As expected, compared with the control group, pIRES2-LINC00312 PC-9 overexpression group showed more lung metastatic nodules. (Fig. 1c). These results indicate that LINC00312 promotes metastasis in vivo. We further analyzed the effects of ectopic LINC00312 expression on tube formation of ADC cells (H1299 and PC-9) to gain insight into the potential role of LINC00312 in VM formation (Fig. 1 d, e). Our data revealed that the average VM density in tumor tissues formed in LINC00312 overexpression cells was significantly higher than that in control cells (Fig. 1f).

### Prognostic significance of LINC00312 expression in combination with VM density

**Fig. 2:**
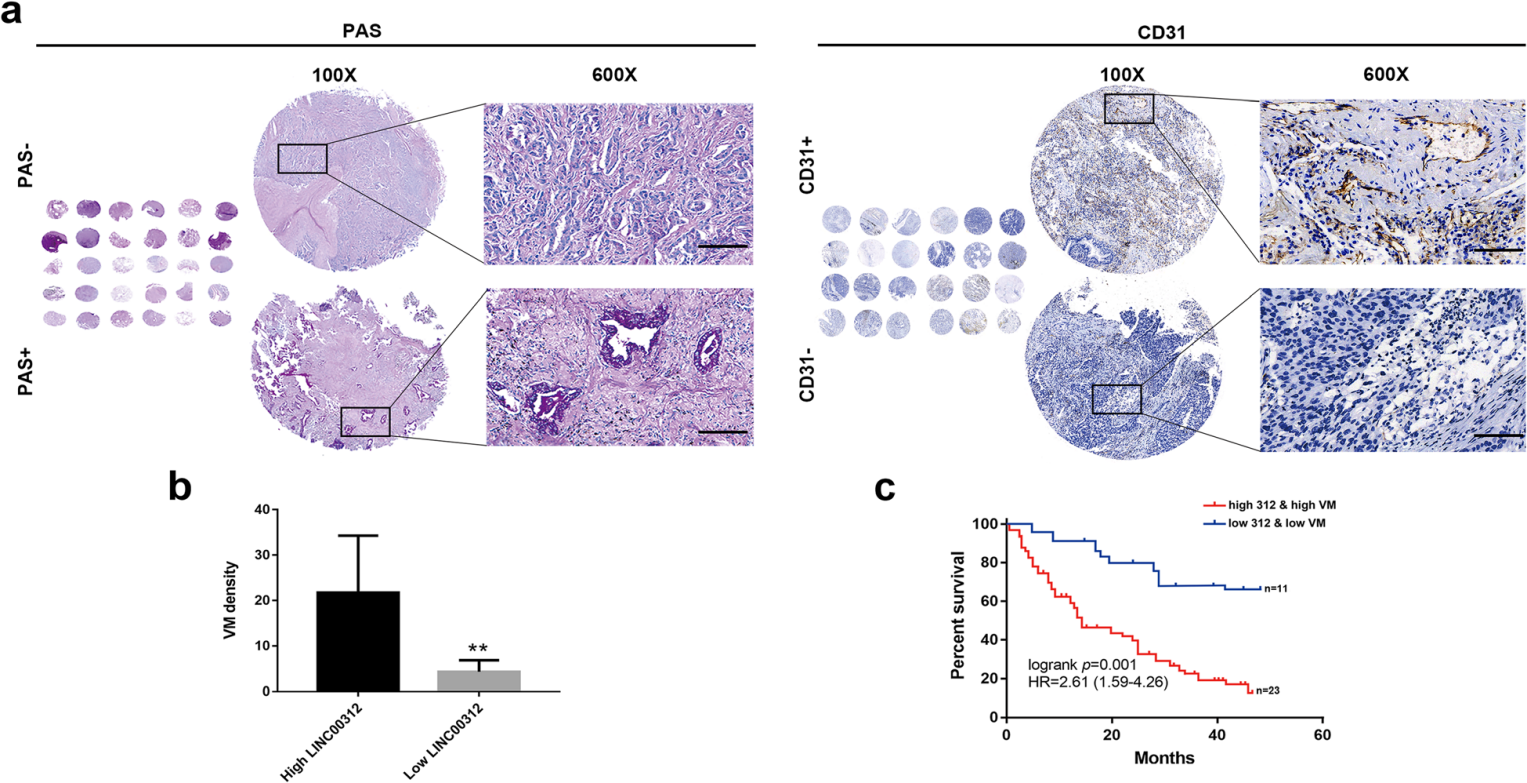
Prognostic significance of LINC00312 expression in combination with VM density. **a** Schematic presentation of typical images of CD31-/PAS+ double staining to explore correlations between LINC00312 and VMs in ADC clinical samples. **b** Quantitative analysis of VMs (CD31-/PAS+). **c** Kaplan–Meier analyses of the correlation between LINC00312 levels, VMs density and OS in the ADC patients. Patients showed worse OS when grouped by high LINC00312 expression together with high VMs. High VMs: VMs density ≥10.2; Low VMs: VMs density ≤10.2. Scale bars: 100μm in 600 x images

Next, we performed CD31 and PAS double staining to explore correlations between LINC00312 and VM in 124 ADC clinical samples. The LINC00312 expression levels and clinicopathological analyses are summarized in Additional file 1: Table S1. We used CD31-/PAS+ as a criterium for VM [5]. Angiogenesis in malignant tumor tissues is associated with a poor clinical outcome in ADC patients, and the patients with VM exhibited greater tumor metastasis and a lower survival rate. Representative images of VM were shown in Fig. 2 a, b and quantified in Additional file 1: Table S1. Patients with simultaneous high levels of LINC00312 expression and high density of VM exhibited a relatively poor prognosis (Fig. 2c).

### LINC00312 promotes migration and invasion of ADC cells

VM is mediated by increased migratory and invasive abilities of cancer cells [6]. The positive correlation between LINC00312 levels and VM prompted us to explore the effects of this lncRNA on ADC cell migration and invasion. We examined the expression of LINC00312 in 6 ADC cell lines. LINC00312 expression was high in H1703 and H2009, low in PC-9, and intermediate in H1299, A549, and H1975 (Additional file 2: Figure S1A). We achieved significant overexpression and knockdown of LINC00312 PC-9 and H1299 cells as validated by RT-PCR, respectively. The PC-9 and H1299 variants were used to explore the functions of LINC00312 in lung cancer cells. To elucidate the mechanisms of LINC00312-mediated VM and metastasis of ADC, we performed wound healing (Additional file 2: Figure S1B), transwell migration, and transwell invasion (Additional file 2: Figure S1C) assays in H1299 and PC-9 with LINC00312 overexpression or knockdown, respectively. The results demonstrated significantly increased migration and invasion as a result of LINC00312 overexpression. In accordance, knockdown of LINC00312 reduced migration and invasion of ADC cell lines.

### LINC00312 directly binds to YBX1

**Fig. 3:**
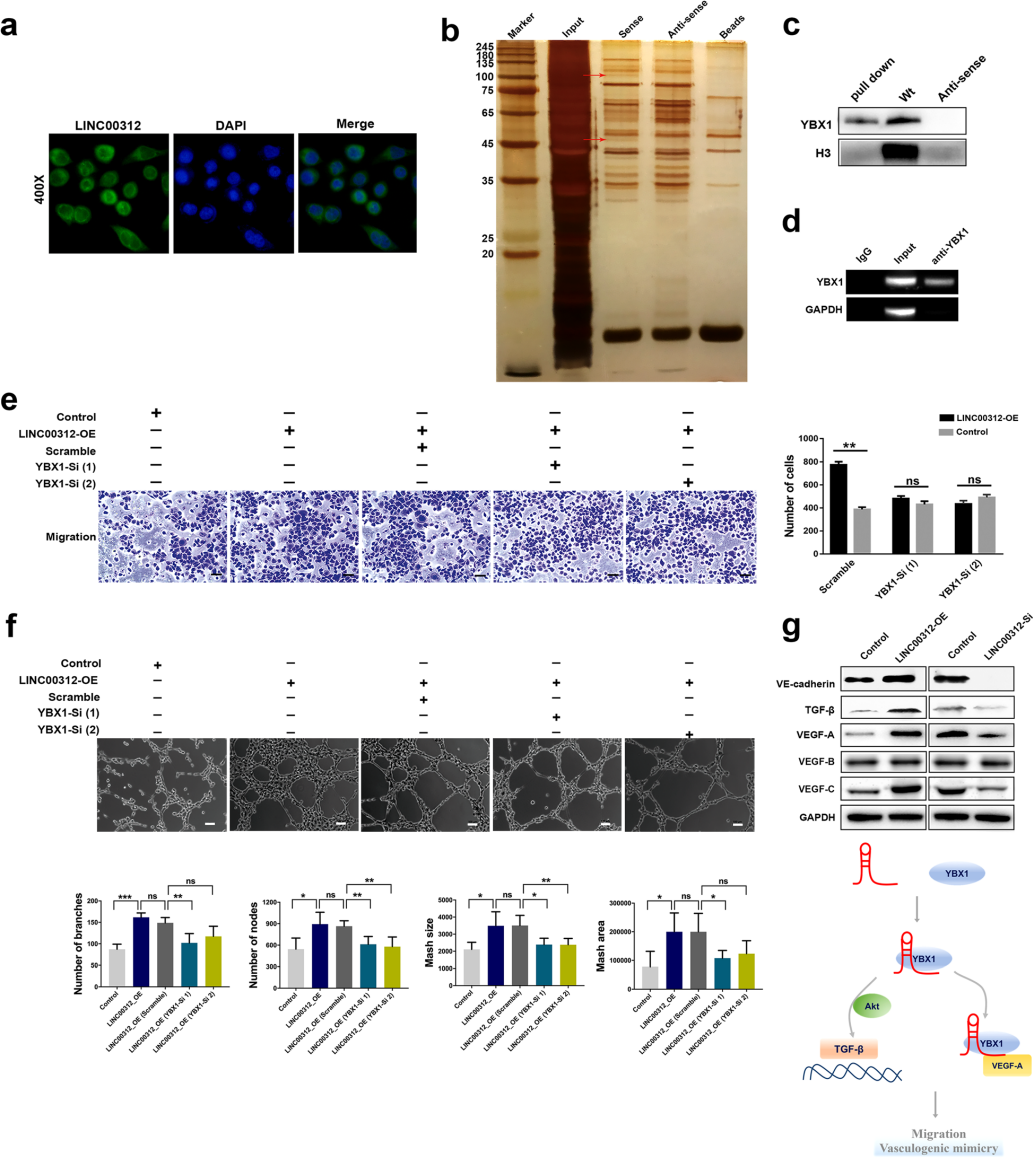
LINC00312 affected angiogenesis and VM formation via YBX1. **a** Fluorescent in situ hybridization analysis of LINC00312 subcellular localization. **b** LINC00312-sense and LINC00312-antisense probes were biotinylated, transcribed in vitro, and incubated with PC-9 whole-cell lysates for RNA pull-down assays. The silver staining represented the two LINC00312–specific bands (red arrows). Schematic repeatedly appeared in three independent assays. **c** LINC00312–specific bands were excised and analyzed by Western blot assay. **d** RIP assays were performed using antibodies against YBX1. Real-time PCR (RT–PCR) were normalized to the housekeeping gene GAPDH. Shown is one representative assay from at least three independent replicates. **e** Transwell migration assays of PC-9 cells transfected with the indicated plasmids. Bar charts showing depleting YBX1 block LINC00312-mediated metastasis. **f** Tube formation assay of VM in PC-9 cells transfected with the indicated plasmids. **g** LINC00312 regulates YBX1-related angiogenic genes of VE-cadherin, TGF-β, VEGF-A, VEGF-B and VEGF-C in PC-9. VE-cadherin, TGF-β, VEGF-A and VEGF-C expression increased markedly following overexpression of LINC00312. VE-cadherin, TGF-β, VEGF-A and VEGF-C decreased following knockdown of LINC00312. Control represent wild cells transfected with vector plasmid. Error bars represent SD from three reactions. Significance was calculated using Student’s t test. *p < 0.05; **p < 0.001; ***p 0.0001; ns, not significant. Scale bars: 100μm in 600 x images

A large number of lncRNAs function through binding to a protein partner. Fluorescent in situ hybridization were performed to examine subcellular localization of LINC00312. The results indicated that LINC00312 was present in the nucleus and cytoplasm (Fig. 3a). To identify the LINC00312 interacting proteins we performed RNA pull-down assay with biotinylated LINC00312, followed by mass spectrometry (MS). Our result indicated that two protein bands were specifically precipitated by LINC00312 in PC-9 in RNA pull-down assay (Fig. 3b). Western blotting was carried out on the protein samples precipitated by the LINC00312 RNA pulldown assays using the antibodies specific for the above candidates. Our results indicated that only YBX1 was precipitated by LINC00312 (Fig. 3c; Additional file 3: Table S2). To further confirm this interaction, we carried out RIP assay using a YBX1-specific antibody, followed by qRT-PCR using the primers specific for LINC00312. As expected, LINC00312 was enriched in the anti-YBX1 group, compared to control IgG group (Fig. 3d). These results indicated that YBX1, a transcription factor that regulates a range of gene expression, directly interacts with LINC00312.

To define the region of LINC00312 that was bound by YBX1, we generated three fragments (LINC00312^I^, LINC00312^II^, LINC00312^III^) according to its secondary structure (Additional file 4: Figure S2A). Pull-down assays demonstrated that YBX1 was enriched by both LINC00312^I^ and LINC00312^II^ (Additional file 4: Figure S2B). Western blotting on the proteins isolated from the RNA pulldown assays was used to determine whether YBX1 interacts with LINC00312^I^ and LINC00312^II^ (Additional file 4: Figure S2C). Western blotting further confirmed the results of pulldown assay. Taken together, these data confirmed that YBX1 binds to LINC00312^I^ and LINC00312^II^of LINC00312.

### LINC00312 affected migration and VM formation via YBX1

A prooncogenic role for YBX-1 is suggested by its ability to promote migration and invasion of tumor cells [6]. Importantly, YBX1 up-regulates pro-angiogenic genes expression and plays a critical role in angiogenic switch [7, 8]. Therefore, we hypothesized that LINC00312 induces migration of ADC cell lines through YBX1. To test this hypothesis, we analyzed migration and invasion when YBX1 was knockdown in the LINC00312 overexpression of PC-9. As expected, YBX1 knockdown partially blocked LINC00312-mediated increase in migration and invasion (Fig. 3e).

Moreover, VM is regarded as an important source for blood perfusion that supplies nutrients and oxygen to tumor growth and promotes cancer metastasis and progression. We postulate that LINC00312 induces ADC cell’s VM via its binding to YBX1. Thus, we examined LINC00312-mediated VM when YBX1 was knockdown by RNAi. As expected, YBX1 knockdown blocked the LINC00312-mediated VM (Fig. 3f). Quantification from three independent replicates is shown in number of branches, nodes, average mesh size area. These data demonstrate that LINC00312 promotes ADC cell’s VM formation via YBX1.

LNC00312 affected many biological processes in ADC cells according to our RNA-seq results. The top GO terms and enriched KEGG pathways are related to YBX1-regulated angiogenic gene expression (Additional file 5: Figure S3).

It has been reported that YBX1 promotes TGF-β-induced migration via Akt activation. Elevated YBX1 expression increases cytosolic localization of VE-cadherin [9]. Moreover, YBX1 affects VEGF-A expression by binding to its promoter [10]. Hence, we further investigated whether LINC00312 affected their expression. As expected, western blot assay demonstrated that LINC00312 significantly increased VE-cadherin, TGF-β, VEGF-A and VEGF-C levels (Fig. 3g).

## Conclusion

We demonstrated that LINC00312 induces lung adenocarcinoma metastasis and VM through direct binding to YBX1. Our study provides a significant advance in the current understanding of the dual roles for LINC00312 in lung cancer.

## Additional files

Additional file 1: **Table S1**. Correlation between LINC00312 expression, VM density and clinicopathological parameters. (XLSX 11 kb)

Additional file 2: **Figure S1**. LINC00312 promotes migration and invasion of ADC cells. (TIF 9436 kb)

Additional file 3: **Table S2**. Proteins associated with biotinylated LINC00312 in RNA pull-down assay. (XLS 425 kb)

Additional file 4: **Figure S2**. A. Secondary structure of LINC00312. B. PC-9 whole-cell lysates pulled down with truncated LINC00312 or antisense probe. C. LINC00312 fragment–specific bands were excised and analyzed by Western blot assay. (TIF 1462 kb)

Additional file 5: **Figure S3**. Functional annotations of differentially expressed genes between LINC00312 stably overexpressed pIRES2-LINC00312 and the vector control PC-9 cells. (A) Schematic presentation of top terms in the GO terms. (B) Schematic presentation of top terms in the KEGG terms. (TIF 4495 kb)

## Abbreviations

lncRNAs: Long non-coding RNAs
MALAT-1: Metastasis-associated lung adenocarcinoma transcript 1
MS: Mass spectrometry
NSCLC: Non-small-cell lung cancer
TNM: Tumor node metastasis
VM: Vascular mimicry
YBX1: Y-Box Binding Protein 1
