# Supplementary figures and images for: The long noncoding RNA LINC00312 induces lung adenocarcinoma migration and vasculogenic mimicry through directly binding YBX1
Source: Mol Cancer. 2018 Nov 23;17:167. doi: 10.1186/s12943-018-0920-z (PMC6260658; doi:10.1186/s12943-018-0920-z)

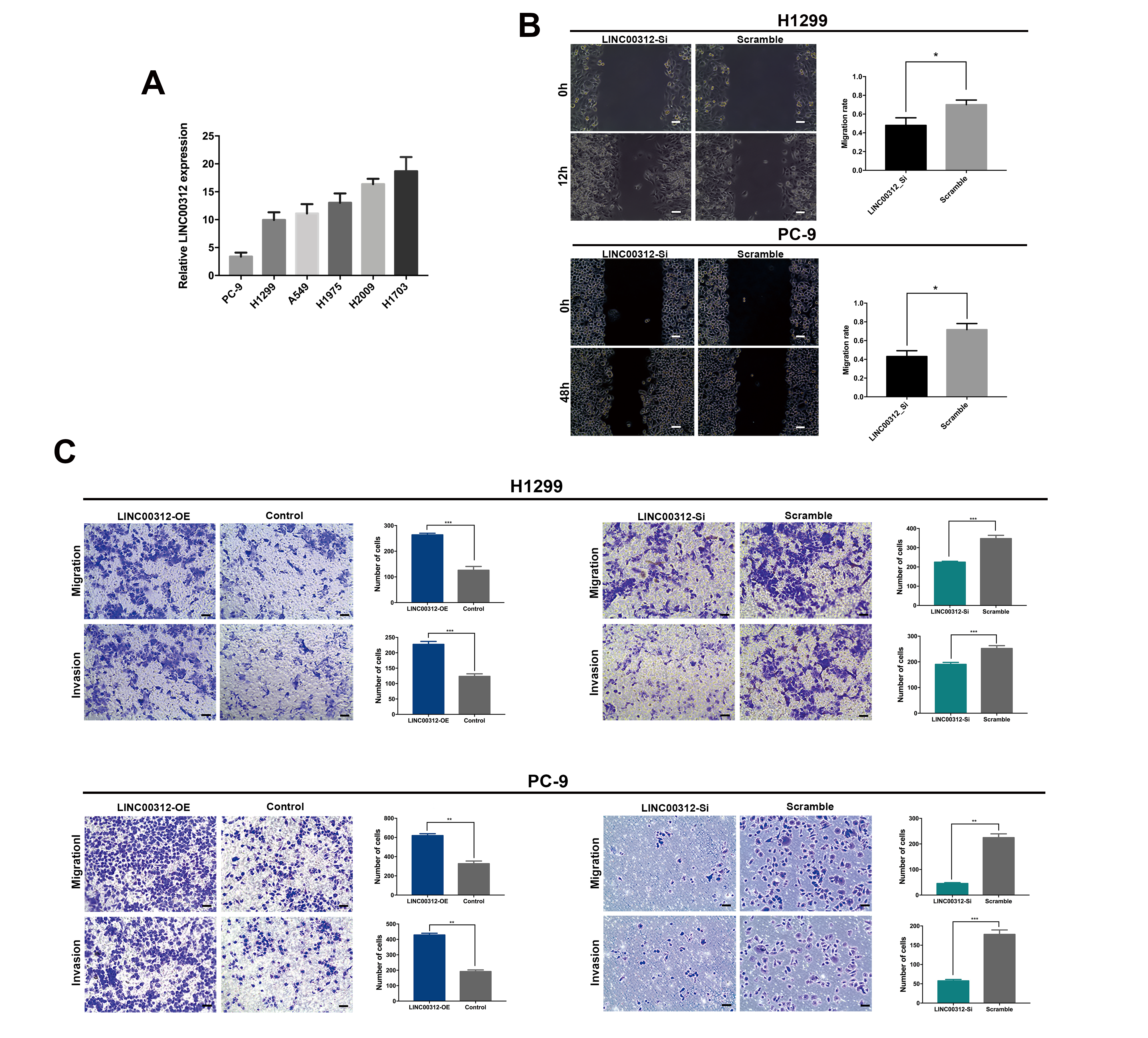

Supplement: Supplementary file 2 — Figure S1. LINC00312 promotes migration and invasion of ADC cells. (TIF 9436 kb) [file 12943_2018_920_MOESM2_ESM.tif]

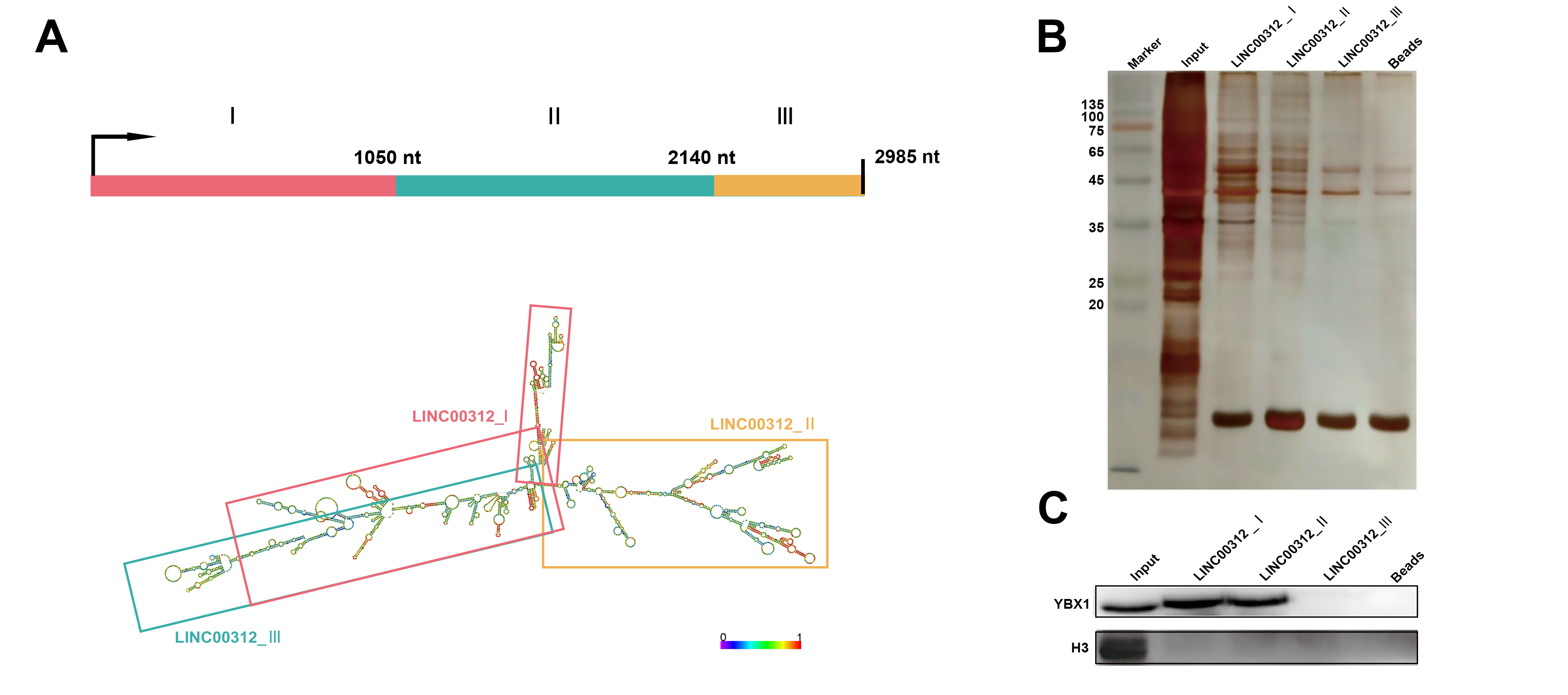

Supplement: Supplementary file 4 — Figure S2. A. Secondary structure of LINC00312. B. PC-9 whole-cell lysates pulled down with truncated LINC00312 or antisense probe. C. LINC00312 fragment–specific bands were excised and analyzed by Western blot assay. (TIF 1462 kb) [file 12943_2018_920_MOESM4_ESM.tif]

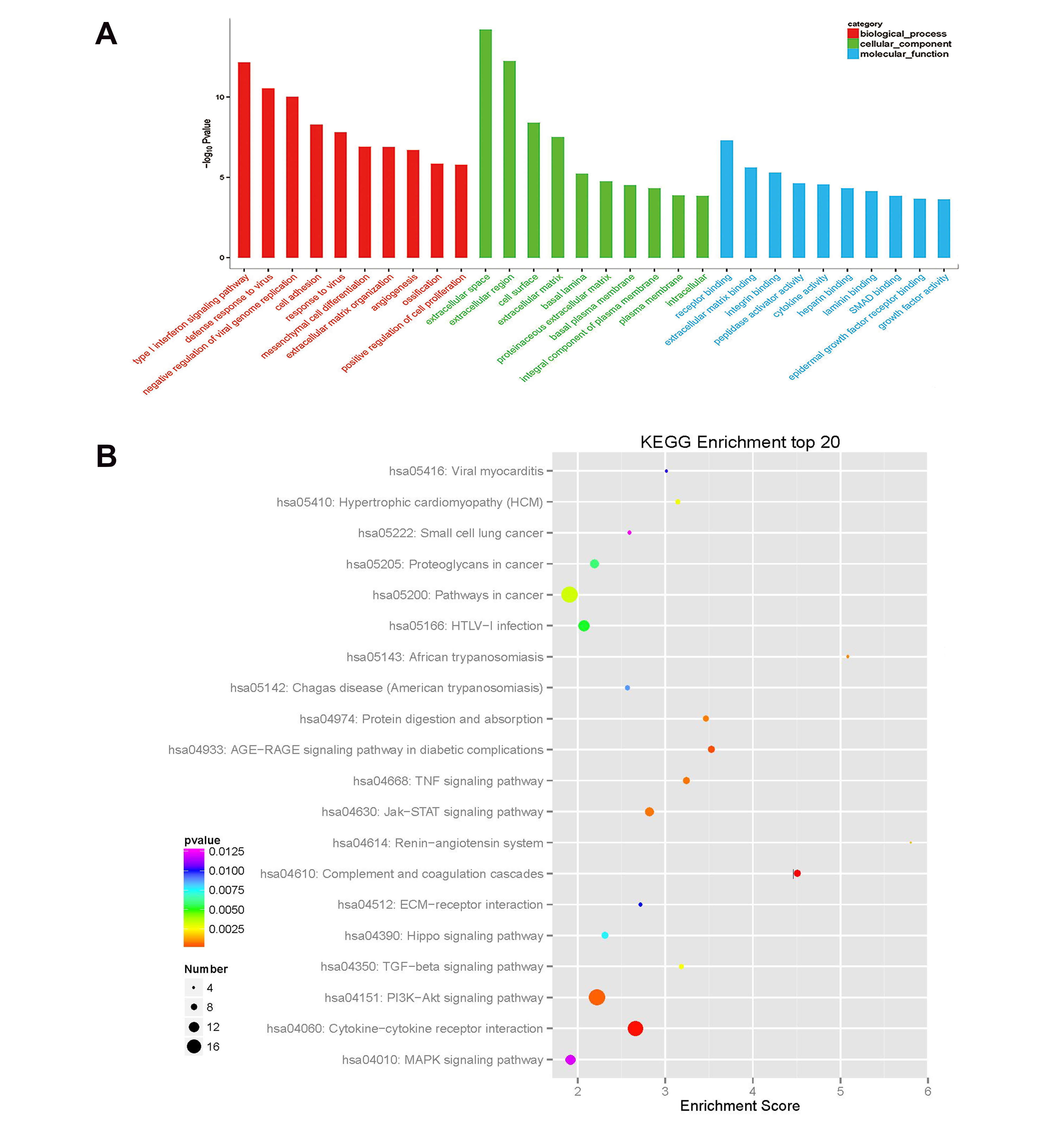

Supplement: Supplementary file 5 — Figure S3. Functional annotations of differentially expressed genes between LINC00312 stably overexpressed pIRES2-LINC00312 and the vector control PC-9 cells. (A) Schematic presentation of top terms in the GO terms. (B) Schematic presentation of top terms in the KEGG terms. (TIF 4495 kb) [file 12943_2018_920_MOESM5_ESM.tif]
